# Distance dependence of near-field fluorescence enhancement and quenching of single quantum dots

**DOI:** 10.3762/bjnano.2.68

**Published:** 2011-09-29

**Authors:** Volker Walhorn, Jan Paskarbeit, Heinrich Gotthard Frey, Alexander Harder, Dario Anselmetti

**Affiliations:** 1 Experimental Biophysics and Applied Nanosciences, Bielefeld University, Department of Physics, Universitätsstr. 25, 33615 Bielefeld, Germany

**Keywords:** AFM, fluorescence energy transfer, multiple multipole simulation, quantum dots

## Abstract

In fluorescence microscopy and spectroscopy, energy transfer processes between single fluorophores and fluorophore quencher pairs play an important role in the investigation of molecular distances or orientations. At distances larger than about 3 nm these effects originate predominantly from dipolar coupling. As these experiments are commonly performed in homogenous media, effects at the interface boundaries can be neglected. Nevertheless, the combination of such assays with single-molecule manipulation techniques such as atomic force microscopy (AFM) requires a detailed understanding of the influence of interfaces on dipolar coupling effects. In the presented work we used a combined total internal reflection fluorescence microscopy (TIRFM)–AFM setup to elucidate this issue. We measured the fluorescence emission emanating from single quantum dots as a function of distance from the apex of a gold-coated cantilever tip. As well as fluorescence quenching at close proximity to the tip, we found a nonlinear and nonmonotonic distance dependence of the fluorescence emission. To confirm and interpret our findings we performed calculations on the basis of a simplified multiple multipole (MMP) approach, which successfully supports our experimental data. Moreover, we revealed and quantified the influence of interfering processes such as field enhancement confined at interface boundaries, mirror dipoles and (resonant) dipolar coupling.

## Introduction

Fluorescence microscopy and spectroscopy are important and versatile tools in life sciences. Fluorophores are not merely position markers, but can be regarded as active transducers that interact with species in their local vicinity and provide information about their micro-environment. The spectroscopic properties of semiconductor nanocrystals (quantum dots) can be easily tuned and they exhibit excellent resistance against photobleaching. Moreover, quantum dots that are functionalized for biological applications are readily available. Locally confined dipole–dipole couplings, such as quenching and fluorescence resonance energy transfer (FRET) [1] between individual molecules, open up fascinating means to explore inter- or intramolecular distances [2], orientation [3], affinity and binding dynamics at the single-molecule level [4]. The combination of fluorescence with single-molecule manipulation techniques, e.g., AFM [5] or optical tweezers [6], opens up novel means of manipulating and controlling matter at the nanometer scale, and also applications such as optomechanics [7] and externally controlled optical switching [8–11]. Nevertheless, surface bound fluorescence assays require solid supports (microbeads, AFM cantilevers, glass substrates, etc.), where fluorophores are not only excited by the incident light, but are also affected by secondary field effects induced at the interface boundaries. Since excited fluorophores polarize their vicinity, they give rise to phenomena such as energy transfer, resonant coupling or shifted angular distribution of fluorescence emission [12–16]. Even though these processes are short ranged, as they predominantly originate from dipole–dipole coupling (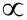
R^−6^), they can significantly affect the observable fluorescence emission. Therefore, a profound knowledge of these effects plays a key role in the acquisition and interpretation of data obtained with combined single-molecule mechano-optical setups.

## Results and Discussion

**Figure 1:**
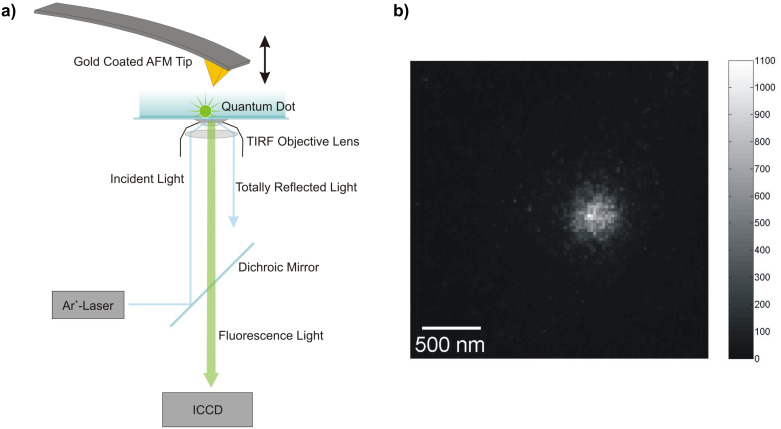
a) Schematic image of the combined TIRFM–AFM setup. The AFM is placed on top of an inverted microscope. The subnanometer spatial resolution of the AFM piezo drive allows precise positioning relative to the sample surface. The incident laser is directed towards the sample surface at an angle of total reflection. The intensity of the evanescent wave projecting beyond the cover slip decreases exponentially. An image-intensified CCD camera detects the fluorescence light. b) Single CCD camera frame of a single quantum dot.

The distance dependence of the electrodynamical coupling between a single dipole emitter located near an air–glass interface and a gold coated AFM cantilever tip was elucidated by means of a combined TIRFM–AFM approach based on a home-built AFM setup that was mounted on an inverted microscope (Figure 1a). The cantilever position relative to the sample surface can be set and adjusted with subnanometer precision. The sample is irradiated by a p-polarized Ar^+^-Laser at an angle of total reflection, resulting in an evanescent wave constrained close to the surface [17]. The fluorescence emission emanating from the sample is detected by an image-intensified charge-coupled device (ICCD) camera (Figure 1b).

**Figure 2:**
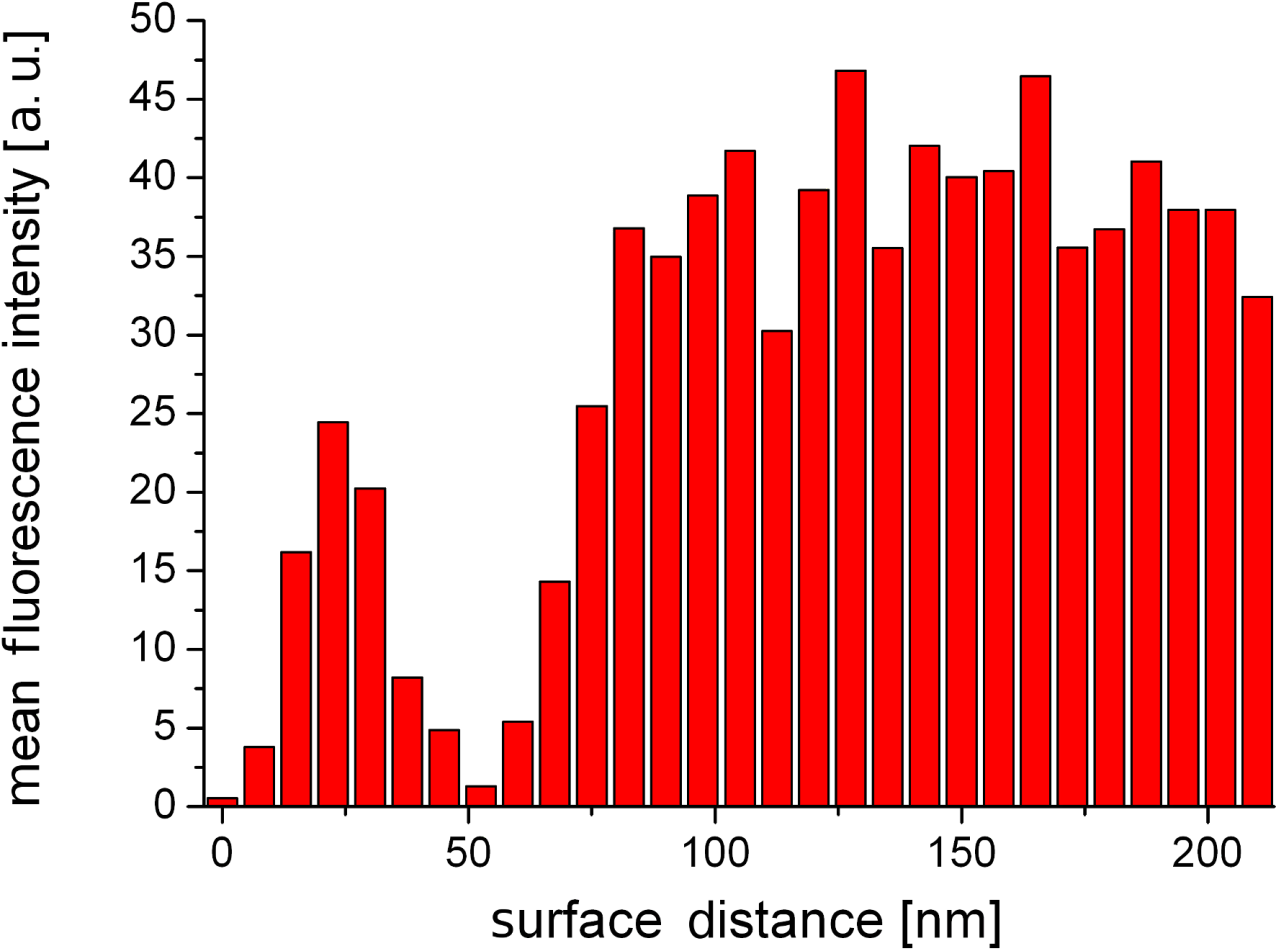
Integrated fluorescence intensity of a single quantum dot as a function of tip distance. The bin size is 7.5 nm (3 × step size of 2.5 nm).

To investigate the dependence of the fluorescence emission from a single quantum dot on the distance from the gold coated cantilever tip apex, we acquired the fluorescence emission intensity at several *z*-distances. After each 2.5 nm step, 200 frames with an exposure time of 50 ms were obtained. The measurements suffer from the typical intermittent fluorescence emission of quantum dots, often referred to as blinking, but the effect on the results was reduced by binning three distance steps together. The integrated fluorescence intensity shows a pronounced distance dependence for gap sizes below 75 nm (Figure 2).

At close proximity to the surface (<20 nm) we found a distinct decrease of the fluorescence emission, which was completely quenched at surface contact. This finding is attributed to energy transfer between the fluorophore and the gold tip. Analogous results for colloidal gold nanoparticles and organic dye molecules were obtained and discussed recently [13]. At larger gap sizes we observed a significant distance dependence of the fluorescence emission. A relative fluorescence maximum at *z* ≈ 22 nm was followed by a drop of the emission intensity at a surface distance of approximately 52 nm. Further retracting led to a recovery of the fluorescence emission until the impact of the tip became negligible. Our experimental findings are well supported by the ensemble data of Govorov et al. [18] who studied the coupling between colloidal gold nanoparticles and CdTe quantum dots coupled by polymer linkers as a function of linker length. The enhanced fluorescence as well as the second minimum must be explained on the basis of two interfering processes caused by the nonhomogeneity of the medium. Firstly, we consider the coupling between the cantilever tip and the evanescent sample illumination: The dipolar coupling between the incident light and the gold tip leads to a field enhancement confined at the tip apex. Secondly, we have to consider the dipolar coupling between the fluorophore and the tip, which either leads to fluorescence enhancement due to resonant coupling or fluorescence quenching as a result of energy transfer. Unfortunately, these effects cannot be observed separately. Hence, to gain a detailed insight into the contribution of the involved processes, we performed multiple dipole (MDP) calculations at tip distances from 5–500 nm. Furthermore, several tip shapes with opening angles ranging from 18–46° were modelled. We evaluated the observable intensity of the fluorescence emission *I* as a function of tip distance, in a three step procedure. Firstly, we examined the interaction between the cantilever tip apex and the incident light. The relative excitation rate Γ_exc_ (Equation 1) of a single dipole emitter that is oriented perpendicular to the sample surface was estimated for several tip distances. Secondly, to evaluate the relative quantum yield *Q* (Equation 2), one has to separate the impact of the coupling between the dipole emitter and the tip apex from that of the secondary fields. Therefore, we calculated the observable fluorescence emission *I* (Equation 3) of a single dipole emitter with Γ_exc_ = constant. Thirdly, to approximate the experimental data, the distance dependence of Γ_exc_ was considered when computing *I*.

**Figure 3:**
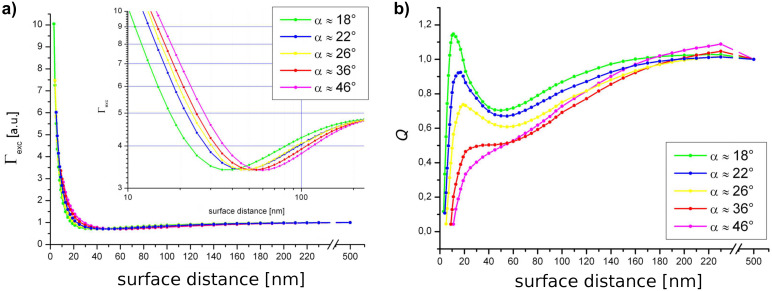
Separated contributions to the external control of fluorescence emission, for several different tip shapes. a) Relative excitation rate Γ_exc_ (inset: semilog plot) at an illumination angle of 45°. b) Relative quantum yield calculated for constant excitation.

**Figure 4:**
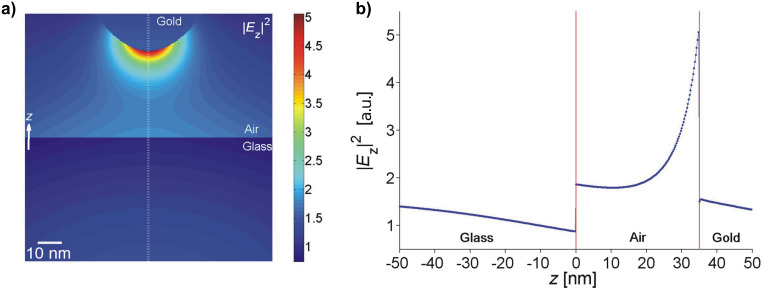
a) Distribution of the field intensity |*E_z_*|^2^ near an evanescently illuminated gold tip 35 nm above an air–glass interface. b) Field intensity |*E_z_*|^2^ along the symmetry axis.

We found a nontrivial dependence of the coupling between the evanescent field and the AFM cantilever tip (Figure 3a). At small tip distances a strong field enhancement is observed that rapidly decreases with growing gap size. This strong distance dependence is characteristic of dipole–dipole coupling effects. Upon further retraction from the surface Γ_exc_ exhibits a relative minimum at tip distances between 35 and 60 nm. The extent of the drop is moderate (approx. 25%) for all tip shapes. Notably, the minimum occurs at smaller surface distances for sharp tips. This effect is likely to stem from interference between mirror dipoles in the glass induced by the strong field confined at the tip apex (Figure 4). Further withdrawing of the tip successively diminishes the impact of such tip-induced effects.

The electrodynamic coupling between a dipole emitter and the tip at constant Γ_exc_ is presented in Figure 3b.

**Figure 5:**
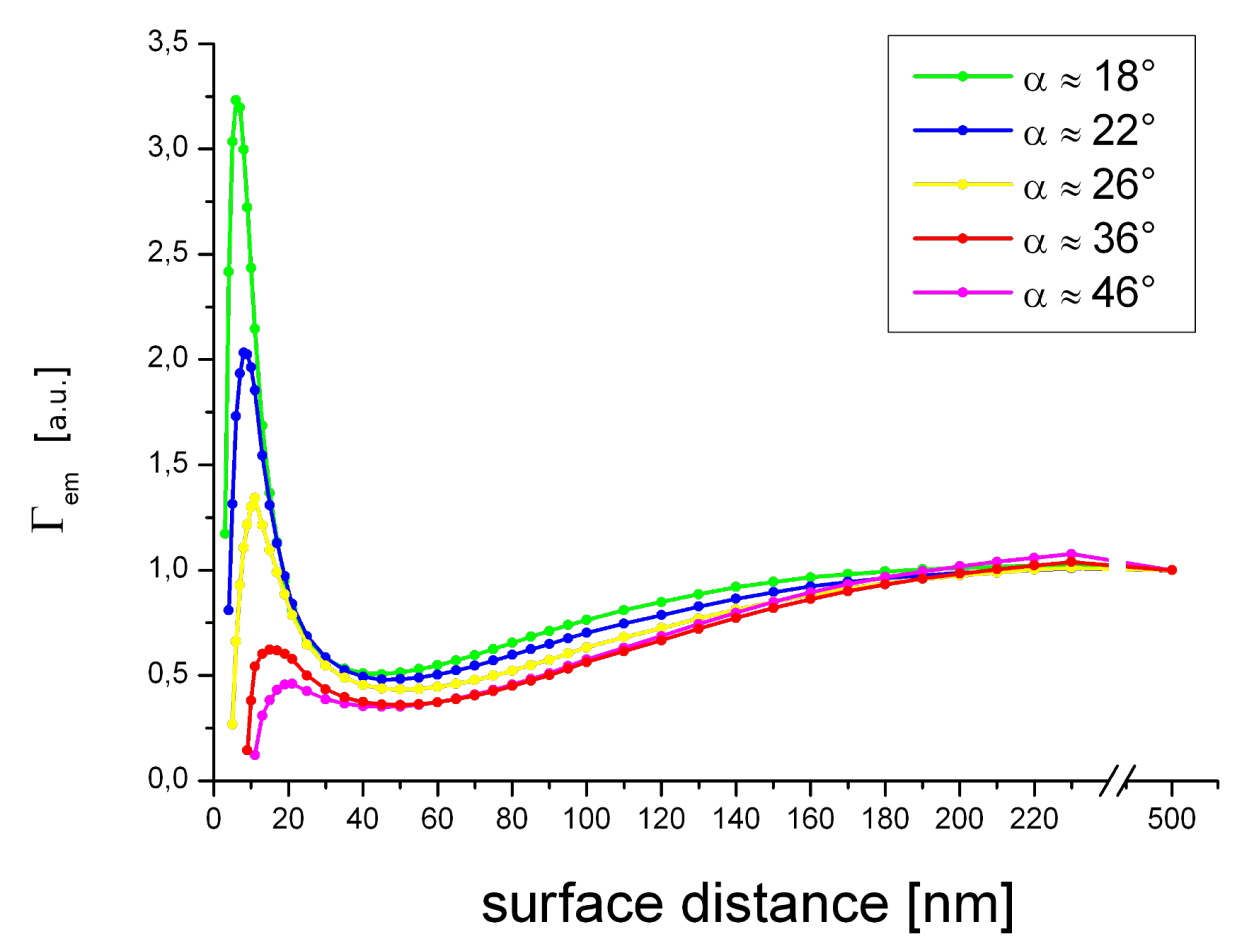
Relative emission rate for several surface distances and tip opening angles estimated by the observable fluorescence emission intensity *I*.

We observe almost complete fluorescence quenching at close proximity to the surface, which is in excellent agreement with recent findings [12–13]. The fluorescence emission, however, does not increase monotonically as the tip is withdrawn from the surface. Instead, we observed an enhanced fluorescence emission at approximately 20 nm, which is followed by partial fluorescence quenching at a gap size of about 50–60 nm. Both effects become less pronounced for larger cone angles. Equally, fluorescence enhancement as well as quenching can be attributed to (resonant) exciton–plasmon coupling. To obtain the observable fluorescence emission *I*, we now consider the variability of fluorophore excitation (Figure 5). Comparison of the theoretical results with our experimental data shows a very good agreement. The surface distances for fluorescence enhancement as well as partial quenching were validated.

Discrepancies between experimental and theoretical data are most likely due to the assumed simplifications. More elaborate approaches that use higher orders of the multipole expansion or lower symmetry may give more precise results. Generally, with our comparably simple model we were able to validate the experimental results qualitatively. Moreover, we were also able to separate and quantify the influence of the enhanced field confined to the tip apex and the impact excitation plasmon coupling on the detectable fluorescence intensity.

**Figure 6:**
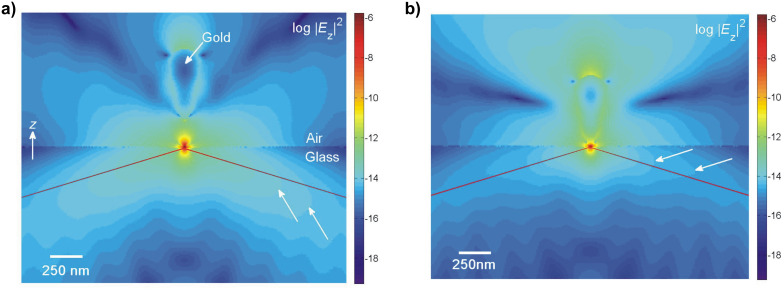
Logarithmic field distribution of a single dipole emitter perpendicular to a glass–air interface for tip distances of a) 200 nm and b) 5 nm. The tip opening angle is 18°. The microscope objective lens detection angle (red) illustrates the change in the angular emission pattern at different tip distances. The direction of highest emission intensity (arrows) shifts to larger angles at smaller tip distances.

Furthermore, we found a considerable shift in the angular distribution of the fluorescence emission (Figure 6) induced by the coupling between the tip apex and the dipole emitter.

**Figure 7:**
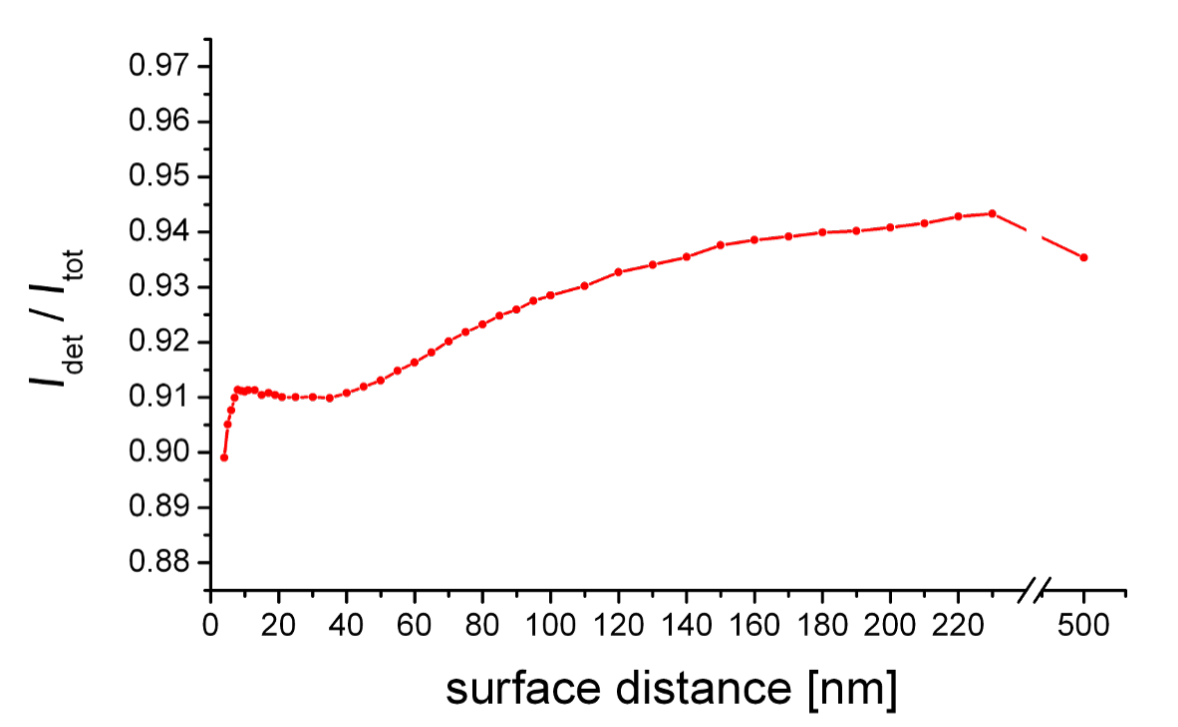
Detection efficiency of an objective lens (numerical aperture (NA) = 1.45) as a function of tip distance.

The angle of highest emission intensity (arrows) lies within the detection angle of the objective lens for the retracted tip, however, it successively shifts beyond the detection angle for decreasing gap size. Consequently, the observed quenching of the fluorescence intensity is not only due to an absolute reduction of the emission rate, but also because of the successive decrease of the detection efficiency 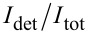 of the objective lens (Figure 7). This finding is well supported by recently published experimental and theoretical data [16]. Yet, in our case the impact of this relative fluorescence quenching is negligible.

## Conclusion

We presented experimental data and simulations for the fluorescence emission control of single quantum dots by the external intervention of a gold-coated AFM tip. The acquired luminescence data exhibited a nontrivial dependence on the tip distance. Modelling the system with a MDP approach unveiled the multivalent interplay of incident and emitted electromagnetic fields at the boundary of different media.

Our results represent an important step en route towards being able to controllably address and manipulate fluorescently labelled individual molecules. Furthermore, the MDP approach is very well suited for qualitative ad hoc validation of experimental data. The significance of dipolar coupling in single molecule manipulation assays was demonstrated. Conceivable applications range from microarrays to controlled manipulation of single molecules. The strong distance dependence of dipole–dipole coupling combined with the subnanometer resolution of AFM holds great promises to yield as yet unattainable information about the interplay of individual molecules, such as their molecular recognition mechanisms [19–24], folding pathways [25] or micro environments [26].

## Experimental

### TIRF–AFM Setup

All experimental work was performed on a combined total internal reflection fluorescence microscopy (TIRFM) atomic force microscope (AFM) setup. The homebuilt AFM head is mounted on an inverted Microscope Axiovert 100 (Carl Zeiss, Oberkochen, Germany) with a high numerical aperture objective lens (Olympus Plapo 100× TIRFM, NA = 1.45, Olympus, Tokyo, Japan). Fluorescence detection was performed by a liquid-cooled image-intensified charge-coupled device (ICCD) camera (I-PentaMAX, Roper Scientific, Trenton, NJ USA). Fluorescence excitation was achieved by an Ar^+^-laser (continuous wave, 10 mW, 488 nm). For excitation power control, neutral density filters with optical density from 0.3–1.5 were installed in the laser path. A detailed description of the setup was published recently [27–28].

The cantilever was approached to the surface in 2.5 nm steps. After each step a series of 200 images was acquired with an exposure time of 50 ms. During the approach to the surface, the cantilever deflection was sampled. The exact tip surface distance was evaluated by linear approximation of the free and contact regimes of the deflection versus piezo extension plot.

### Sample and cantilever preparation

Microscope glass cover slips (24 × 24 mm^2^, Menzel, Germany) were washed with acetone, ethanol and water and dried gently with nitrogen. To remove any fluorescent adsorbates, the substrates were dipped in boiling piranha solution (1:3 sulphuric acid 96% and hydrogen peroxide 30%) for one minute, rinsed thoroughly with MilliQ filtered water and dried with nitrogen. After cleaning, hydrophobic fluorescent CdSe/ZnS nanoparticles [29] with a spectral emission maximum at 585 nm were diluted in *n*-heptane (Sigma Aldrich), microdispensed to the glass cover slips and dried. Sparsely covered (<1 quantum dot per 25 µm^2^) samples allowed the addressing of individual fluorophores.

Silicon AFM cantilevers (PPP-NCHR, Nanosensors, Neuchatel, Switzerland) were washed with acetone, ethanol and water and dried in a gentle flow of nitrogen. Subsequently, a 50 nm thick gold layer was evaporated on the cantilevers at a rate of 0.2 nm/s.

### Multiple dipole (MDP)-Simulation

To calculate the electromagnetic field distribution near interface boundaries one has to solve Maxwell’s equations with regard to the boundary conditions. These are derived in common electrodynamics textbooks [30]. As our system is more complex than planar interfaces, analytical approaches are meaningless. Therefore, we selected a simplification of the multiple multipole (MMP) method [31–32], which is a semi-analytical approach to compute field distributions in arbitrarily shaped piecewise homogeneous, isotropic and linear media. In brief: The electromagnetic fields at the domain boundaries are approximated numerically whereas the field strength within the domain can be computed analytically. The system is modelled by choosing a set of matching points on the domain boundary. Multipole emitters along each side of the interface induce an electromagnetic field exclusively in the opposing domain. The strength of each emitter is approximated numerically in such a way that the boundary conditions are satisfied at the matching points. Superposition of all multipole emitter contributions results in a field distribution that is a solution of Maxwell’s equations and satisfies the boundary conditions. To limit the demand for processing power and memory we made some simplifications: Firstly, we assumed cylindrical symmetry (along the *z*-axis). The sample is evanescently illuminated by p-polarized light leading to an enhancement of the field component normal to the interface. Thus, the polarization beyond the surface is almost parallel to the *z*-axis. The contribution parallel to the surface can therefore be neglected. Secondly, we omitted the silicon–gold interface. In principle, our tip surface geometry can be compared to a Kretschman–Raether configuration [33]. Even though this model is only applicable to planar geometries, it can serve as an ad hoc approximation for our more complex system. Consequently, the surface plasmon decay length *z* perpendicular to the boundary surface can be approximated by the following expression:

[4]
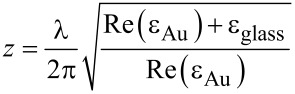


where λ denotes the wavelength, and ε_Au_ and ε_glass_ the dielectric functions of gold and glass, respectively. With the corresponding dielectric functions (see below) we determined a plasmon decay length of *z* ≈ 59 nm, which is of the order of the thickness of the gold layer on the tip. Hence, the influence of the silicon tip core should be insignificant. Thirdly, we only considered the contribution of dipole emitters to the field distribution and omitted higher orders. Consequently, this approach will be referred to as multiple dipole (MDP) method.

MDP calculations were performed for various tip shapes and surface distances. As quantum mechanical effects such as electron transfer are not considered in this classical approach, the minimum tip surface distance was set to 5 nm. The tip geometry was modelled by cones with opening angles in the range of 18–46°; as tip apex a second order polynomial was appended continuously. The angle of sample illumination was set to 45°, which is well above the critical angle of total reflection (approx. 43°) at an air–glass interface. Corresponding to the experiment, the wave lengths for illumination and fluorescence emission were set to 488 nm and 585 nm, respectively. The dielectric functions ε of the corresponding medium at the given wavelengths are: ε_air_(488 nm) = ε_air_(585 nm) = 1, ε_glass_ (488 nm) = 2.34, ε_glass_ (585 nm) = 2.33, ε_Au_ (488 nm) = −1.33 + 3.06i and ε_Au_ (585 nm) = −7.7 + 1.06i [34].

In order to quantify the fluorophore excitation, we computed the relative excitation rate Γ_exc_ (Equation 1), which we define as the quotient of the excitation rate of the undisturbed system 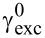 (surface distance >500 nm) and the excitation rate γ_exc_ in proximity to the cantilever tip.

[1]
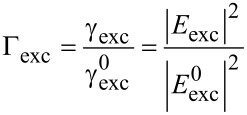


In terms of electric field strength, this can be expressed as the quotient of the corresponding field intensities at the location of the fluorophore. Accordingly, we define the relative emission rate Γ_em_ (Equation 2) of a single fluorophore by the product of Γ_exc_ and relative quantum yield *Q*.

[2]
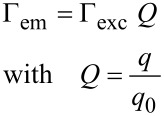


where *q* and *q*_0_ are the apparent and intrinsic quantum yield, respectively. Generally, the shift of the quantum yield can be described in terms of the radiative and nonradiative decay rates (γ_r_, γ_nr_) as follows:

[3]
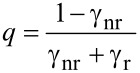


The coupling between a dipole emitter and a sharp metallic tip results in an increase of γ_r_ [32,35]. Yet, the degree of luminescence enhancement is inherently limited by the fluorophore’s intrinsic quantum yield *q*_0_, i.e., strong luminescence enhancement can only be observed for low *q*_0_ (γ_nr_ >> γ_r_).

In order to quantify *Q*, namely the impact of dipolar coupling between the gold tip apex and the fluorophore in the absence of any secondary fields, the detectable fluorescence emission *I* for arbitrary but constant Γ_exc_ was calculated. The normalized intensity of the electric field distribution 
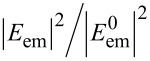
 propagating in the lower glass half-space was integrated over a sphere (Equation 3). To rule out the contribution of the nonpropagating near-field, the sphere radius was set to *R* = 1000 µm. The integration limit of the polar angle Θ_max_ is given by the numerical aperture (NA = *n*∙sin Θ_max_) of the objective lens. Consistent with the experiment we assumed a refractive index *n* = 1.51, and NA = 1.45.

[5]
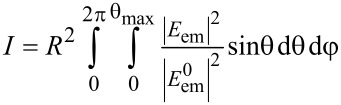


The approximation of the experimental data was made analogously, however, the distance dependence of Γ_exc_ was taken into consideration.
